# Engineering of *Yarrowia lipolytica* transporters for high-efficient production of biobased succinic acid from glucose

**DOI:** 10.1186/s13068-021-01996-w

**Published:** 2021-06-27

**Authors:** Zhennan Jiang, Zhiyong Cui, Ziwei Zhu, Yinghang Liu, Ya-jie Tang, Jin Hou, Qingsheng Qi

**Affiliations:** 1 State Key Laboratory of Microbial Technology, Shandong University, Binhai Road 72, Qingdao, 266237 People’s Republic of China; 2 CAS Key Lab of Biobased Materials, Qingdao Institute of Bioenergy and Bioprocess Technology, Chinese Academy of Sciences, Qingdao, 266101 People’s Republic of China

**Keywords:** *Yarrowia lipolytica*, Transporter engineering, Succinic acid, Glucose fermentation

## Abstract

**Background:**

Succinic acid (SA) is a crucial metabolic intermediate and platform chemical. Development of biobased processes to achieve sustainable SA production has attracted more and more attention in biotechnology industry. *Yarrowia lipolytica* has a strong tricarboxylic acid cycle and tolerates low pH conditions, thus making it a potential platform for SA production. However, its SA titers in glucose media remain low.

**Results:**

In this study, we screened mitochondrial carriers and C4-dicarboxylic acid transporters to enhance SA secretion in *Y. lipolytica*. PGC62-SYF-Mae strain with efficient growth and SA production was constructed by optimizing SA biosynthetic pathways and expressing the transporter SpMae1. In fed-batch fermentation, this strain produced 101.4 g/L SA with a productivity of 0.70 g/L/h and a yield of 0.37 g/g glucose, which is the highest SA titer achieved using yeast, with glucose as the sole carbon resource.

**Conclusion:**

Our results indicated that transporter engineering is a powerful strategy to achieve the efficient secretion of SA in *Y. lipolytica*, which will promote the industrial production of bio-based SA.

**Supplementary Information:**

The online version contains supplementary material available at 10.1186/s13068-021-01996-w.

## Background

Succinic acid (SA) is a vital C4 platform chemical, which has a wide range of applications in food and chemical engineering [1–3]. SA is the main raw material for the synthesis of biodegradable plastic polybutylene succinate and polybutylene succinate adipate [4, 5]. Reforming the production method and increasing the supply of SA is crucial in addressing the high-cost production of biodegradable material. The petrochemical process has numerous disadvantages, including large energy consumption, serious environmental pollution, and the use of nonrenewable fossil resources. Therefore, eco-friendly processes for the production of biobased SA (bio-SA) have attracted more and more attention [3, 6]. However, bio-SA has a higher price tag than petrochemically produced SA because of the low production efficiency of industrial strains and costly downstream processes [7–9].

*Yarrowia lipolytica* is an unconventional yeast that attracted considerable attention for its excellent lipid biosynthesis ability. With the development of metabolic engineering and synthetic biology, this yeast has increasingly been applied to other biotechnological fields [10–13]. In 2010, Yuzbashev et al. [14] inactivated succinate dehydrogenase (SDH) subunits and achieved SA accumulation through the oxidative TCA pathway in *Y. lipolytica*. Our group previously constructed an engineered *Y. lipolytica* strain named PGC202 with robust properties by deleting the SDH5 subunit encoding gene *YlSdh5* and the acetyl-CoA hydrolase encoding gene *YlAch*, and overexpressing phosphoenolpyruvate carboxykinase (ScPck) and succinyl-CoA synthase (YlScs) [15–17]. PGC202 produced 110.7 g/L SA with glycerol as a substrate, without controlling the pH during the fermentation. These reports indicated that *Y. lipolytica* could be used as a microbial chassis for the commercial production of bio-SA. However, SDH inactivation is difficult to achieve in aerobic *Y. lipolytica* and leads to cell growth and metabolism disorders in glucose media [14, 18]. Therefore, Yuzbashev et al. [19] isolated an evolved strain Y-4215 that utilizes glucose efficiently to produce 50.2 g/L SA by adopting a combination approach of mutagenesis and metabolic evolution. Similarly, Li et al. [20] developed a metabolic evolution process with an in situ fibrous bed bioreactor, obtained an evolved strain PSA 3.0, which restored the glucose utilization ability. Through fed-batch fermentation, the PSA 3.0 strain achieved an SA titer of 76.8 g/L. An SA-producing strain of *Y. lipolytica* was engineered by truncating the promoter of the *SDH1* gene, which resulted in a 77% reduction in SDH activity, but did not impair the growth ability of the strain on glucose [21]. This strain produced 35.3 g/L SA at pH 5.5. However, aerobic production of SA in *Y. lipolytica* with glucose as a carbon source presented numerous limitations, and thus, this approach requires further investigation.

**Fig. 1:**
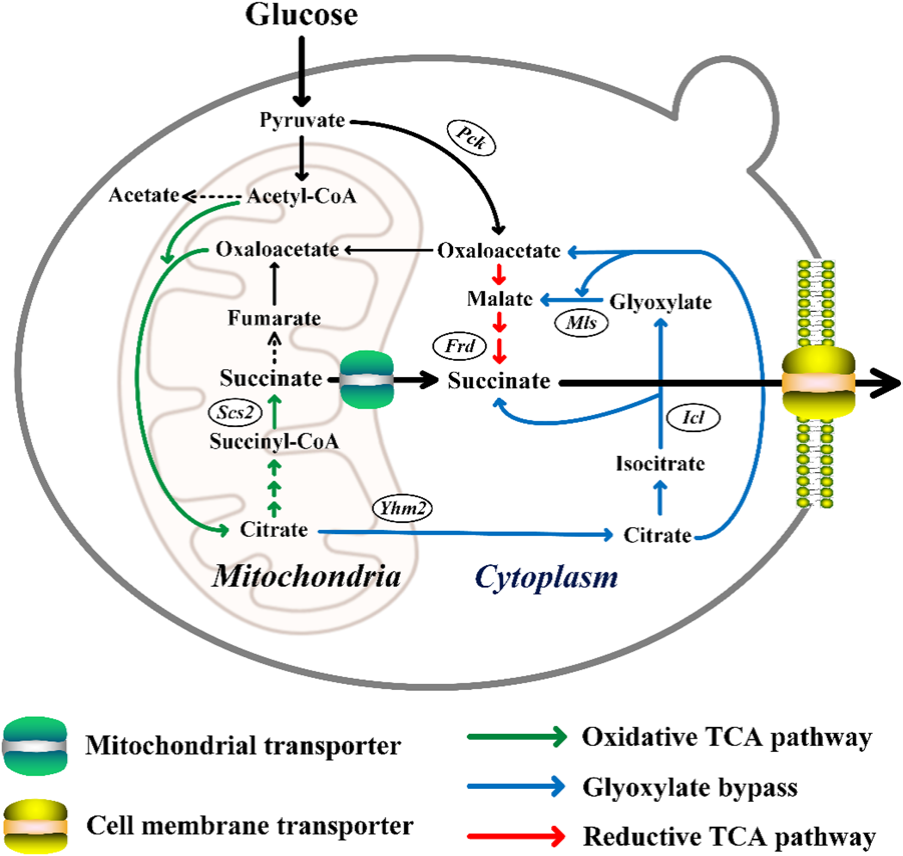
Schematic diagram of the SA biosynthetic pathways and its export route in *Y. lipolytica*. There are three major metabolic pathways involve in the SA biosynthesis of *Y. lipolytica*: (1) reductive branch of the TCA cycle (red line); (2) oxidative TCA cycle (green line); (3) glyoxylate bypass (blue line). Dotted lines indicated the deleted genes for SA accumulation. *Pck* phosphoenolpyruvate carboxykinase, *Scs2* succinyl-CoA synthase β subunit, *Frd* fumarate reductase, *Mls* malate synthase, *Icl* isocitrate lyase, *Yhm2* mitochondrial citrate transporter

Transporter engineering is an effective approach to improve substrate utilization and product secretion from microbial cell factories [22, 23]. Enhancing the efflux of the final product can not only reduce feedback inhibition and cellular toxicity but also shift the reaction equilibrium, thereby facilitating downstream steps. The main location for SA biosynthesis in *Y. lipolytica* is the mitochondrial matrix. Therefore, SA must be transported across the inner mitochondrial membrane and cell membrane to achieve extracellular secretion (Fig. 1). The insufficient rate of SA transportation may be a key factor limiting the efficiency of bio-SA production by *Y. lipolytica*. In this study, we identified the endogenous mitochondrial transporters responsible for SA efflux and screened heterogenous C4-dicarboxylic acid transporters using an SA-producing strain of *Y. lipolytica*, PGC62. A chassis cell with an optimized SA biosynthetic pathway was then constructed to improve SA production and evaluate the function of SA transporters. The SA synthesis and secretion by *Y. lipolytica* were simultaneously enhanced to increase the efficiency of bio-SA production from glucose.

## Results and discussion

### Identification of the transporters for mitochondrial efflux of SA in *Y. lipolytica*

Mitochondrial carriers (MCs) are localized in the inner membranes of mitochondria and mediate the exchange of metabolites, such as organic acid, amino acids, nucleotides, and cofactors, between the mitochondrial matrix and cytoplasm [24–26]. In the *Y. lipolytica* genome DNA, 39 genes were predicted to encode MCs [27, 28]. A mitochondrial citrate carrier, YlYhm2, was identified in *Y. lipolytica*, and its expression caused a substantial increase in citric acid production [28]. No specific mitochondrial succinate transporter has been identified. Five MCs that were hypothesized to transport carboxylic acid were selected and overexpressed in the engineered strain PGC62, which is an previously constructed *Y. lipolytica* strain for SA production [15], to promote the efflux of SA across the inner mitochondrial membrane. As illustrated in Table 1, all expressed strains were associated with higher SA production than the control. Overexpression of the mitochondrial succinate–fumarate transporter YlAcr1 and mitochondrial 2-oxodicarboxylate carrier YlOdc1 increased SA titer by 21.3% and 19.7%, respectively. The highest SA titer and yield of 23.6 g/L and 0.62 g/g glucose, respectively, was observed in the PGC62-YlDic strain.

**Table 1:** Expression of the mitochondrial carboxylic acid transporters in *Y. lipolytica*

RefSeq	Putative coding product	Strain name	DCW (g/L)	SA titer (g/L)	SA yield (g/g glucose)
YALI0F26323g	Mitochondrial citrate transporter	PGC62-YlCtp1	9.5 ± 0.16	20.8 ± 0.15	0.57 ± 0.04
YALI0F20966g	Mitochondrial tricarboxylate transporter	PGC62-YlCtp2	9.8 ± 0.61	20.5 ± 0.25	0.53 ± 0.01
YALI0B03344g	Mitochondrial dicarboxylate transporter	PGC62-YlDic	9.0 ± 0.04	23.6 ± 3.50	0.62 ± 0.12
YALI0D02629g	Mitochondrial 2-oxodicarboxylate carrier	PGC62-YlOdc	9.4 ± 0.21	21.9 ± 2.05	0.52 ± 0.05
YALI0E34672g	Mitochondrial succinate-fumarate transporter	PGC62-YlAcr	8.3 ± 1.35	22.2 ± 0.6	0.59 ± 0.01
N/A	N/A	PGC62	8.3 ± 0.02	18.3 ± 0.39	0.57 ± 0.03

**Fig. 2:**
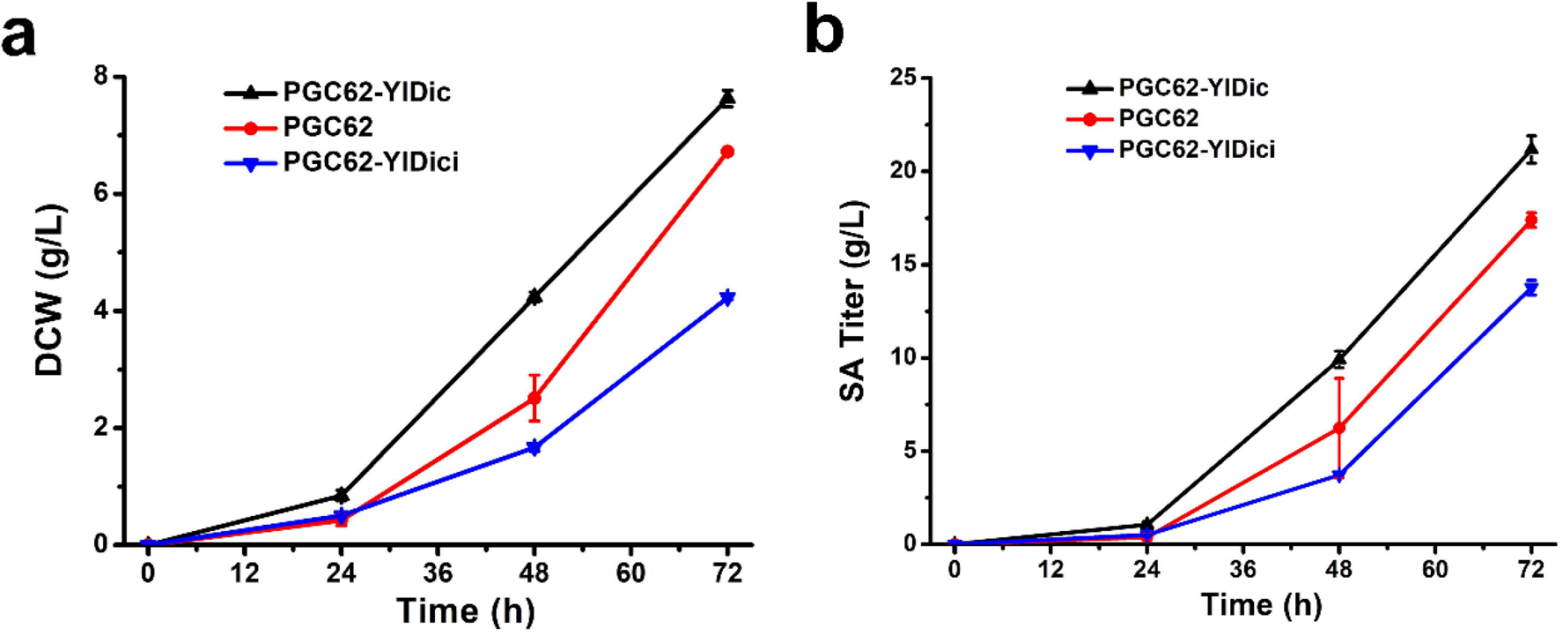
Effect of overexpression and suppression of *YlDic1* gene on the cell growth and SA production of *Y. lipolytica* PGC62 strain. PGC62-YlDic was derived from PGC62 strain with overexpressed *YlDic1* gene. PGC62-YlDici was derived from PGC62 strain with down-regulated *YlDic1* gene through CRISPRi method. Error bars show the SDs of 3 biological replicates

To confirm these findings, the *YlDic1* gene of the PGC62 strain was knocked down using the CRISPR interference (CRISPRi, Additional file 1: Figure S1) method to obtain PGC62-YlDici, and the results were compared with those of PGC62 and PGC62-YlDic. As illustrated in Fig. 2, the DCW and SA titer of PGC62-YlDici decreased by 58.9% and 26.4% compared with PGC62 strain, reached 4.2 g/L and 13.8 g/L, respectively. Suppression of *YlDic1* impaired cell growth and SA production, whereas the *YlDic1* expressing strain displayed a more favorable fermentation performance than PGC62 (Fig. 2). These results indicated that the mitochondrial dicarboxylic acid transporter YlDic1 plays a crucial role in the efflux of succinate from mitochondria, and other MCs may also participate in this process.

### Screening C4-dicarboxylic acid transporters in *Y. lipolytica* to enhance the extracellular SA secretion

**Fig. 3:**
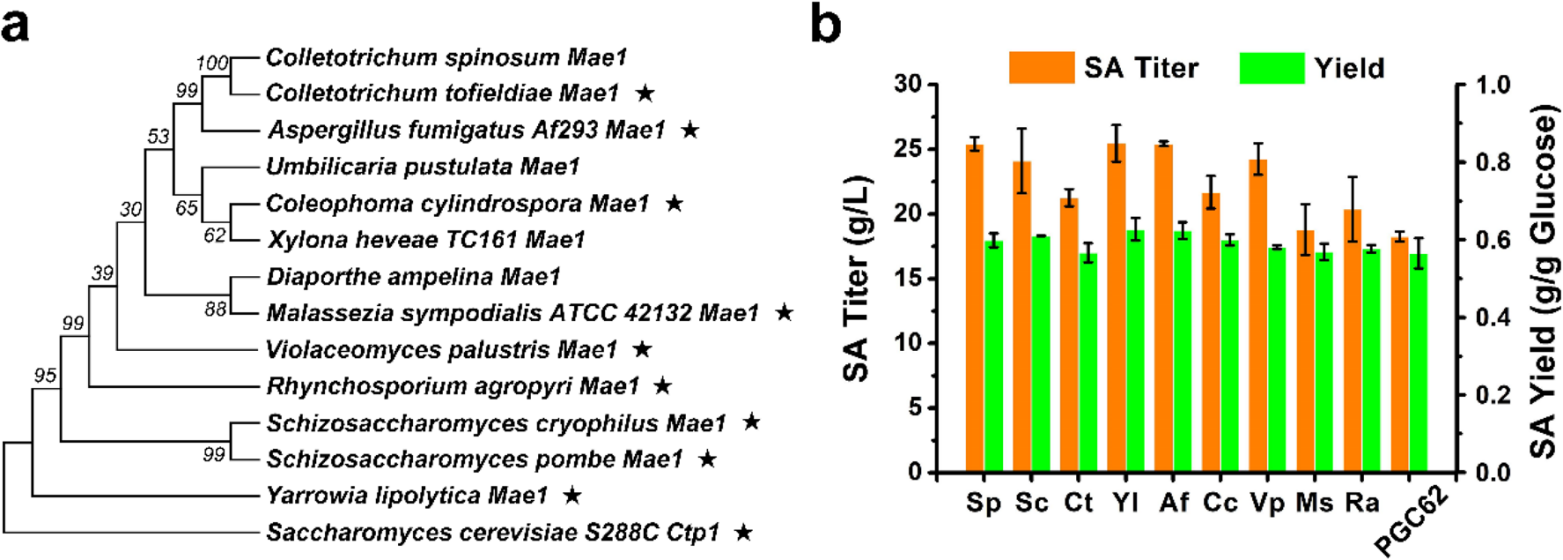
Overexpression of C4-dicarboxylic acid transporters from different species to improve the SA production of *Y. lipolytica*. **a** Nine potential C4-dicarboxylic acid transporters were selected according to the phylogenetic tree; **b** effect of the different C4-dicarboxylic acid transporters on the SA production of *Y. lipolytica*. Error bars show the SDs of 3 biological replicates

Mae1 is a C4-dicarboxylic acid transporter that likely belongs to the voltage-dependent slow-anion channel transporter (SLAC1) family. Mae1 has been applied to increase the secretion of malate and succinate in several studies [29–31]. We examined several C4-dicarboxylic acid transporters from different eukaryotic microorganisms to screen the C4-dicarboxylic acid transporter that can specifically export succinate to the outside of *Y. lipolytica*. We used the well-studied SpMae1 from *Schizosaccharomyces pombe* as a reference to construct a phylogenetic tree based on amino acid sequence similarity and selected candidate transporters (Fig. 3a). The selected transporters were then overexpressed in the PGC62 strain. As illustrated in Fig. 3b, overexpression of SpMae1, YlMae1, AfMae1, ScMae1, and VpMae1 improved the SA titer of PGC62 (increased above 33%), whereas overexpression of CtMae1, CcMae1, MsMae1, and RaMae1 had little effect on SA production. The SA titer of endogenous YlMae1 expressing strain increased by 39.3% compared with the control. YlMae1 (encoded by YALI0E24167p) was initially identified as a sulfite pump, and our results indicated that YlMae1 may be a crucial transporter for the native secretion of SA in *Y. lipolytica*. As studies have reported, SpMae1 does not use proton motive force and is thus less energetically expensive than the majority of other dicarboxylic acid transporters [32]. The SA titer of PGC62-SpMae was increased to 25.2 g/L. These results demonstrated that C4-dicarboxylic acid transporters from different species can enhance the extracellular secretion of SA in *Y. lipolytica*. Among these transporters, SpMae1 has been widely applied to improve the microbial production of malate, succinate and fumarate [30, 31, 33], thus it can be used in the subsequent modification to obtain the SA over-production strains.

### Construction of a chassis cell with optimized SA biosynthetic pathways

**Fig. 4:**
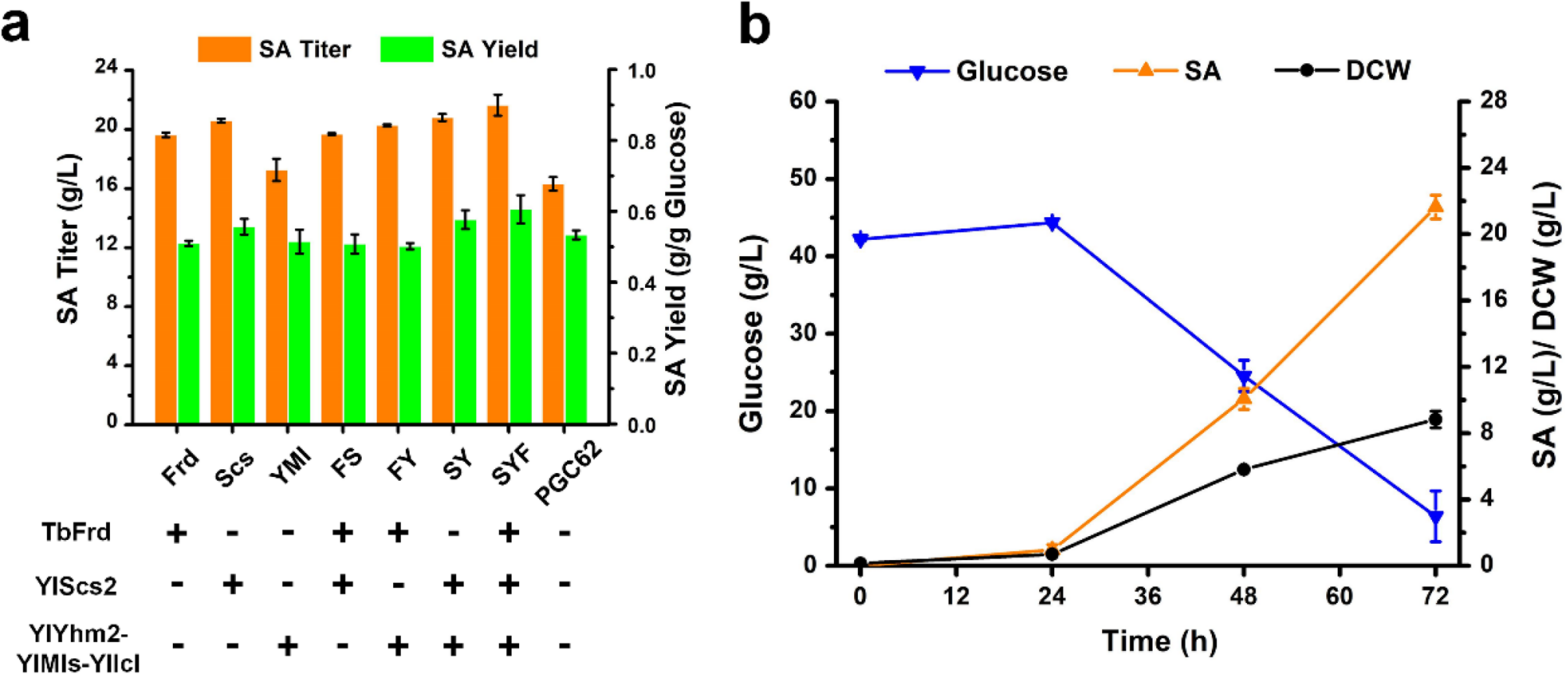
Construction of the *Y. lipolytica* chassis cell through optimizing SA biosynthetic pathways. **a** Effect of the different genetic modifications on SA production of *Y. lipolytica*. To enhance the metabolic flux of reductive TCA, oxidative TCA and glyoxylate pathway, the expression cassettes of *TbFrd*, *YlScs2* and *YlYhm2*-*YlMls*-*YlIcl* were overexpressed in PGC62 strain, respectively; **b** fermentation profile of the engineered strain PGC62-SYF with optimized SA biosynthetic pathway in shaking flasks. Error bars show the SDs of 3 biological replicates

To achieve high SA production and compare the function of transporters, a *Y. lipolytica* chassis was constructed by optimizing the expression of SA biosynthetic pathways. First, the fumarate reductase encoding gene *TbFrd* from *Trypanosoma brucei* [34, 35] was expressed to enhance the carbon flow of the reductive TCA bypass (Additional file 1: Figure S2a). Second, the expression library of the succinyl-CoA synthetase β subunit encoding gene *YlScs2* was constructed to increase the flux through the oxidative TCA pathway (Additional file 1: Figure S2b). Third, endogenous isocitrate lyase YlIcl, malate synthase YlMls, and mitochondrial citrate transporter YlYhm2 [28] were overexpressed to enhance glyoxylate bypass. As illustrated in Fig. 4a, the SA titer of oxidative TCA, reductive TCA, and glyoxylate pathway enhanced strains increased by 26.2%, 20.3%, and 6%, respectively, compared with the starting strain PGC62. The theoretical conversion rate was considerably higher than that of oxidative TCA, because the reductive TCA pathway was not accompanied by CO_2_ release [2]. However, the SA yield of the *TbFrd* overexpression strain PGC62-Frd was only 0.51 g/g glucose, which indicated that establishing a reductive TCA pathway was difficult for the strictly aerobic strain *Y. lipolytica*. The combination of oxidative TCA and glyoxylate bypass resulted in a further increase in the SA titer to 20.8 g/L. Finally, a chassis strain of PGC62-SYF with higher SA production capacity was constructed through the simultaneous expression of three SA biosynthetic pathways. The final SA titer and yield of the PGC62-SYF strain increased by 32.6% and 13.5% compared with PGC62, reaching 21.6 g/L and 0.61 g/g, respectively (Fig. 4). These results indicated that the expression optimization of SA biosynthetic pathway is a useful strategy to obtain the chassis cell with enhanced SA production.

### SpMae1 is crucial for cell growth and SA production in glucose media

**Fig. 5:**
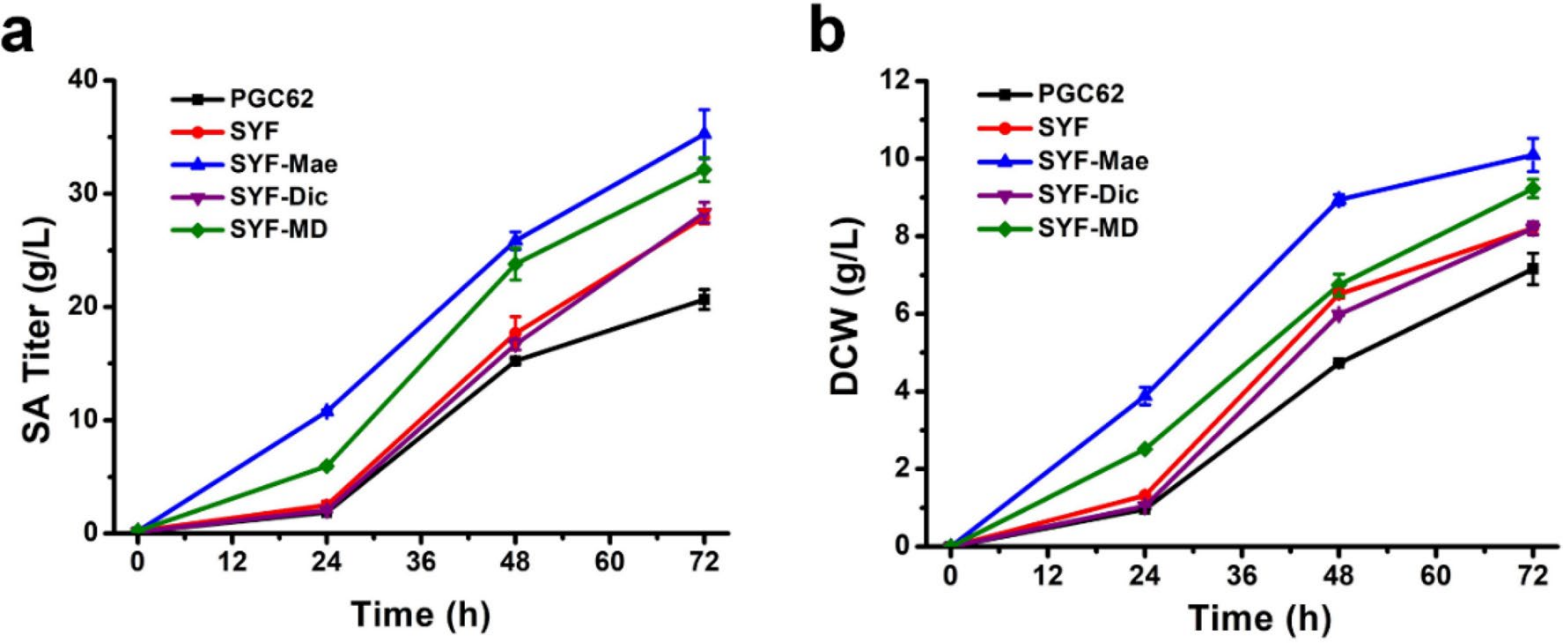
Comparison of the fermentation profiles between different SA producing *Y. lipolytica* strains. **a** Differences in SA production performance of different engineered strains. **b** Differences in the cell growth of different engineered strains. Error bars show the SDs of 3 biological replicates

The engineered strain PGC62-SYF was used as chassis to evaluate the SA transporters’ function. The cell membrane transporter SpMae1 and mitochondrial transporter YlDic1 were expressed in the PGC62-SYF strain to obtain PGC62-SYF-Mae and PGC62-SYF-Dic, respectively. PGC62-SYF-Mae produced 35.3 g/L SA during shaking flask fermentation, which was 26.5% and 16.5% higher than the PGC62-SYF and PGC62-SpMae (Fig. 5a and Additional file 1: Figure S3). The SA titer of PGC62-SYF-Dic was only 28.3 g/L, with no improvements compared with the control strain. As illustrated in Fig. 5b, the growth rate of PGC62-SYF-Mae displayed clear advantages over other engineered strains, with its biomass reaching 10.1 g/L DCW after 72 h of cultivation. The mitochondrial dicarboxylic acid transporter YlDic1 was then introduced into the PGC62-SYF-Mae strain, and combined overexpression of mitochondrial and cellular transporters displayed reduced SA production (Fig. 5a). The results indicated that the SA production those engineered strains were closely related to the cell growth.

YlDic1 was not the only mitochondrial transporter responsible for the SA efflux. Overexpression of YlDic1 cannot further improve the SA production of PGC62-SYF, which indicated that the efficiency of SA transport across the inner mitochondrial membrane was sufficient in *Y. lipolytica*. SA is an acidic compound, and thus, its excessive accumulation is toxic to the hosts. Moreover, the export of organic acids is typically proton-coupled or sodium-coupled and requires energetic expenditure [22], which may be one of the main reasons for the glucose metabolism disorders of SDH-deficient *Y. lipolytica* strains. The overexpression of SpMae1 minimizes feedback inhibition and SA toxicity because of its energy efficiency [31, 36]. In sum, enhancing the SA biosynthetic pathway and transmembrane transport can alleviate the cell growth and metabolism inhibition of the SA-producing *Y. lipolytica* strain in glucose media.

### Fed-batch fermentation for highly efficient SA production from glucose

**Fig. 6:**
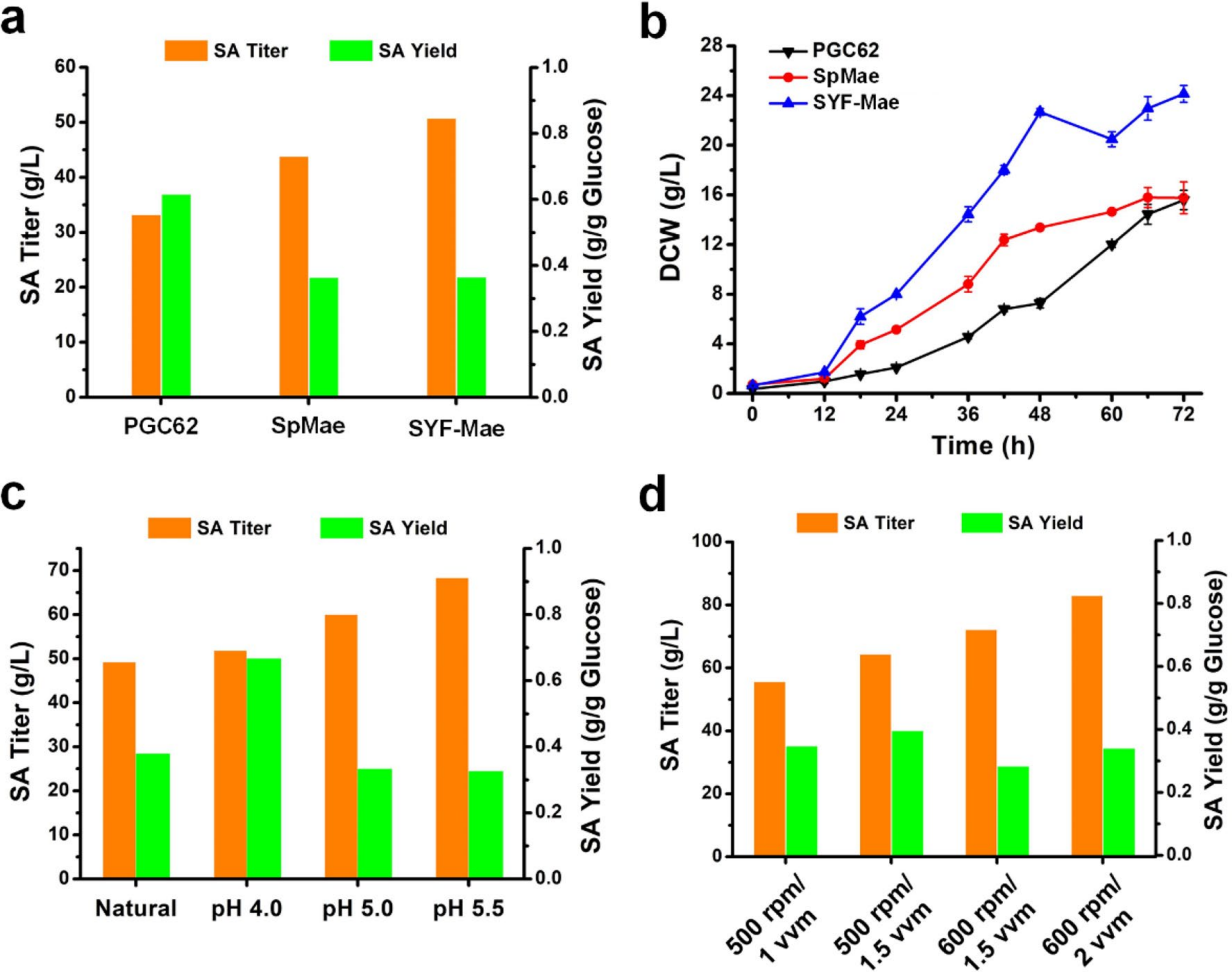
Optimization of culture conditions for SA over-production in 1 L bioreactor. **a** Comparison of the SA production performance between different *Y. lipolytica* engineered strains; **b** growth curves of different *Y. lipolytica* engineered strains; **c** effect of the pH values on the SA production of PGC62-SYF-Mae strain; **d** effect of the air flows and stirring rates on the SA production of PGC62-SYF-Mae strain. Error bars show the SDs of 3 technical replicates

To evaluate the SA production potential of the PGC62-SYF-Mae strain, fed-batch fermentation with glucose as the sole carbon resource was performed in the bioreactor. Similar to the results of shaking flask fermentation, the maximum SA titer was obtained in the PGC62-SYF-Mae strain (Fig. 6a). In terms of cell growth, PGC62-SYF-Mae reached a stable period after 48 h of cultivation with a rapid growth rate, which is similar to SDH-deficient *Y. lipolytica* strains that using glycerol as carbon source [37]. The dry cell weight of PGC62-SYF-Mae was 24.1 g/L after 72 h (Fig. 6b). Overexpression of SpMae1 enhanced the cell growth rate. The pH values and dissolved oxygen levels were then investigated to identify the optimal conditions for SA production. The pH value of the fermentation broth without adjustments was approximately 3.9 at the end of the cultivation, and its SA titer only reached 49.6 g/L. Under pH 5.5, the highest SA titer was 68.39 g/L at 120 h (Fig. 6c). Based on these findings, different stirring rates and air flows were set: 500 rpm/1.0 vvm, 500 rpm/1.5 vvm, 600 rpm/1.5 vvm, and 600 rpm/2.0 vvm. Figure 6d illustrates that the accumulation of SA continuously improved as the airflow and stirring rates increased. Along with the rapid growth of the PGC62-SYF-Mae strain, the final SA productivity reached 0.7 g/L/h. High dissolved-oxygen conditions were conducive to efficient SA production in *Y. lipolytica*, which accords with previous reports [15].

**Fig. 7:**
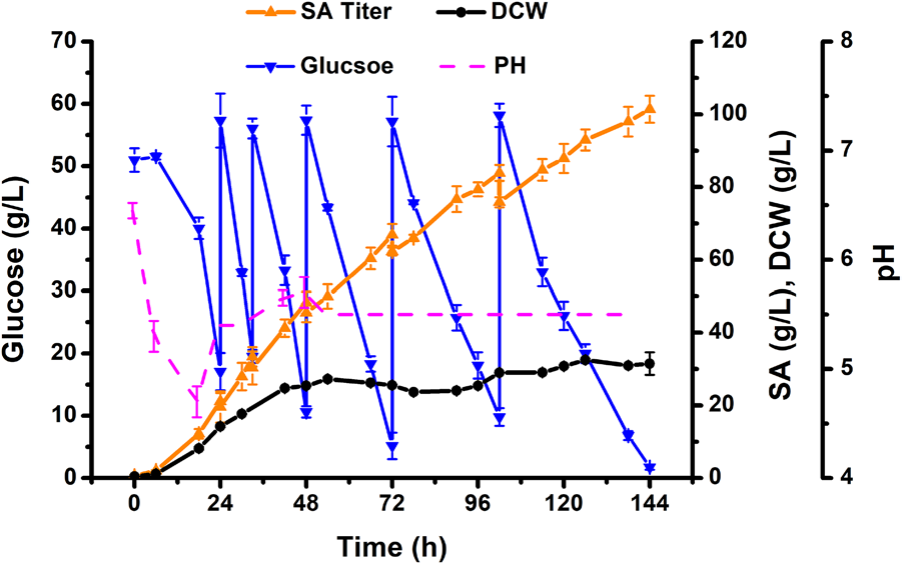
Kinetics of cell growth, glucose consumption and SA production during fed-batch culture of *Y. lipolytica* engineered strain PGC62-SYF-Mae in bioreactor. Error bars show the SDs of 3 biological replicates

Under optimal conditions (pH 5.5, airflow rate of 2 vvm, stirring speed of 600 rpm, and initial glucose concentration of 50 g/L), the fermentation period was extended to 138 h (Fig. 7). The biomass increased rapidly in the first 48 h and reached 30.9 g/L DCW, with a high glucose consumption rate. In total, 263.2 g/L of glucose was consumed for SA production, and the highest SA titer was 101.4 g/L. To our knowledge, this is the highest SA production achieved by eukaryotic microorganisms to date with glucose as the sole carbon source (Table 2). The average SA productivity and yield were 0.70 g/L/h and 0.37 g/g glucose, respectively. These results suggested that the engineered strain PGC62-SYF-Mae can achieve efficient glucose utilization and SA production and thus could be used as a microbial cell factory for the commercial production of bio-SA.

**Table 2:** Comparison of SA production by yeasts with glucose as sole carbon source

Strain	Fermentation condition	SA titer (g/L)	SA yield (g/g glucose)	SA productivity (g/L/h)	References
*S. cerevisiae* SUC-297	Dual phase fed-batch, synthetic medium, pH 5.0 to pH 3.0	43.0	N/A	0.45	[38]
*S. cerevisiae* pRS313CF	Feb-batch, synthetic medium, pH 3.8	12.97	0.14	0.11	[39]
*Pichia kudriavzevii* 13723	Batch, synthetic medium, pH 3.0	48.2	0.45	0.97	[35]
*Zygosaccharomyces rouxii* V19	Batch, YPD medium, pH 5.0	7.7	0.08	N/A	[40]
*Issatchenkia orientalis* SD108	Batch, synthetic medium, neutralization by CaCO_3_	11.6	0.12	0.11	[41]
*Y. lipolytica* Y-4215	Feb-batch, synthetic medium, without pH control	50.2	0.43	0.93	[19]
*Y. lipolytica* Y-3753	Repeated-batch, synthetic medium, without pH control	55.3	0.34	1.15	[14]
*Y. lipolytica* PSA02004	Feb-batch, YPD medium, pH 6.0	65.7	0.50	0.68	[18]
*Y. lipolytica* PSA3.0	Feb-batch, YPD medium, without pH control	76.8	0.20	0.24	[42]
*Y. lipolytica* PGC202	Feb-batch, YPD medium, without pH control	71.6	N/A	0.4	[20]
*Y. lipolytica* PGC202	Feb-batch, YPD medium, without pH control	53.6	0.61	0.49	[17]
*Y. lipolytica* ST8578	Feb-batch, YPD medium, pH 5.0	35.3	0.26	0.61	[21]
*Y. lipolytica* PGC62-SYF-Mae	Feb-batch, YPD medium, pH 5.5	101.4	0.37	0.70	This study

Yeast is an outstanding microbial host for bio-SA production because of its high tolerance to acidic environments [8]. As a model microorganism, *Saccharomyces cerevisiae* possesses a clear genetic background and complete genetic manipulation tools, while SA production in *S. cerevisiae* remains insufficient. Therefore, SA production in several unconventional yeast species, including *Pichia*, *Candida*, *Issatchenkia*, and *Yarrowia*, has been investigated (Table 2). Among them, *Y. lipolytica* is the most studied. The highest SA titer from glucose in the developed yeasts has remained far below the acceptable range for large-scale production. In this study, over 100 g/L SA was produced from glucose using the engineered *Y. lipolytica* strain PGC62-SYF-Mae. However, the SA yield and productivity of the PGC62-SYF-Mae strain are still low. Therefore, further metabolic modification and process optimization should be implemented to improve SA production efficiency.

## Conclusion

This study provided a transporter engineering strategy for the efficient production of bio-SA by *Y. lipolytica* from glucose. We determined that the overexpression of several mitochondrial transporters, including YlDic1 and C4-dicarboxylic acid transporter Mae1, promotes the SA secretion in the *Y. lipolytica* PGC62 strain. By optimizing the flux of SA biosynthetic pathways, the engineered strain PGC62-SYF was constructed to further evaluate the function of SA transporters. Cellular membrane transporter SpMae1 from *S. pombe* was confirmed to be effective in promoting cell growth and SA production in glucose media. The SA titer reached 101.4 g/L in fed-batch fermentation, which is the highest fermented SA titer achieved by yeast hosts with glucose as the sole carbon source. The strain constructed in this work contributes to the development of sustainable and low-cost production methods of bio-SA.

## Materials and methods

### Strain, media, and growth condition

DH5α was grown in Luria–Bertani broth (LB) containing 50 mg/L ampicillin for routine subcloning and plasmid propagation. *Y. lipolytica* PGC62, a succinate production engineered strain derived from Po1f, was constructed previously [15]. PGC62 strain was used for further genetic engineering and its derived strains were presented in Additional file 1: Table S1. *Y. lipolytica* strains were cultured at 30 °C, 220 rpm using YPG media (20 g/L glycerol, 20 g/L tryptone, 10 g/L yeast extract). Synthetic SD media supplemented with suitable amino acid dropout mixes and 20 g/L glycerol was used for the auxotrophic screening of positive transformants. 400 mg/L Hygromycin B (Yeasen, Shanghai, China) and 20 g/L agar were added to YPG and SD media when necessary. All media were sterilized at 121 °C for 20 min, and then were inoculated under sterile conditions.

### Plasmid and strain construction

All restriction enzymes used in this study were purchased from Thermo Fisher Scientific (Shanghai, China). PCR amplifitions were implemented using PrimerSTAR Max DNA Polymerase (TaKaRa, Beijing, China). All heterologous genes were optimized according to the codon preference of *Y. lipolytica* and synthesized by GENERAL BIOL (Anhui, China). Gibson Assembly Cloning Kit (New England Biolabs (NEB), England) [43] was used for plasmid construction. The primers and plasmids used in this study were listed in Additional file 1: Tables S2 and S3, respectively.

The encoding genes of hypothetic C4-dicarboxylic acid transporters derived from *Colletotrichum tofieldiae* (CtMae1, KZL77415), *Aspergillus fumigatus* Af293 (AfMae1, XP_747269), *Coleophoma cylindrospora* (CcMae1, RDW74776), *Malassezia sympodialis* ATCC 42132 (MsMae1, SHO79003), *Violaceomyces palustris* (VpMae1, PWN50824), *Rhynchosporium agropyri* (RaMae1, CZT12766), *Schizosaccharomyces cryophilus* (ScMae1, XP_013022427), and *S. pombe* (SpMae1, NP_594777) were synthesized and cloned into the expression vector pKi-1 (Additional file 1: Table S4). The endogenous genes YlMae1, YlCtp1, YlCtp2, YlDic1, YlOdc1, and YlAcr1 were amplified from the genomic DNA of wild-type *Y. lipolytica* W29 strain, and assembled with the *Bsp119*I-digested pKi-1. YlScs2 was amplified and inserted into the *Bsp119*I-digested pKi-1 to obtain expression vector pKi-1-Scs. *YlYhm2*, *YlIcl*, and *YlMls* were amplified and assembled with 113-GDP-TEF to obtain 113-YIM. Fumarate reductase encoding gene *TbFrd* from *Trypanosoma brucei* was synthesized and cloned into the *Bsp119*I-digested pKi-hyg to construct expression vector pKi-hyg-Frd.

The dCas9 and sgRNA expression cassette was mutated and amplified through overlap PCR using the plasmid pCAS1yl-trp as template. This cassette was then assembled with KpnI-digested JMP114 to obtain the pCRISPRi plasmid. Optimal 20 bp crRNA sequences that located on the frontal region of targeted genes were designed with the help of an online tool (http://chopchop.cbu.uib.no/) [44].

The expression plasmids were then digested by NotI to obtain the integrated DNA fragments. These linearized fragments were transformed into *Y. lipolytica* strains through the lithium acetate method as previously reported [45], to construct the expression library via NHEJ-mediated random genome integration. Positive transformants were selected on appropriate plates and checked by colony PCR. 10 transformants were randomly picked, and the engineered strains with high SA production were screened through shaking flasks fermentation. The plasmid pUB4-Cre for the expression of Cre recombinase was used to recycle the selective markers [46].

### Shaking flasks fermentation

The shaking flasks fermentation were carried out in 250 mL shake flasks with 50 mL YPD media, and the initial concentration of glucose was set as 60 g/L. After inoculation in 2%, cells were cultivated at 30 °C and 220 rpm. Samples were taken every 24 h for measuring optical density, glucose, SA, and other products. All the fermentation conducted in shake flask had not added any alkali to maintain neutral pH condition. All the experiments were run in triplicates, the average and standard deviation values were reported.

### Fed-batch fermentation in bioreactor

The SA producing strains were first inoculated into 5 mL 2% YPG medium and cultured at 30 °C and 220 rpm for 36 h. Culture was inoculated into 80 mL 2% YPG medium in 500 mL shake flasks as seed culture at 30 °C and 220 rpm. Seed culture (50 mL) was then inoculated into modified YPD media (50 g/L glucose, 20 g/L tryptone, 10 g/L yeast extract) to start fed-batch fermentation.

Different cultivation conditions such as pH, air flow and stirring rate were tested in 1 L fermenter (INFORS Multifors Bacteria, Switzerland). The pH was set as nature, 4.0, 5.0 or 5.5. The air flux and stirring rate were set as 1.0/500, 1.5/500, 1.5/600, or 2.0/600 (vvm/rpm). During fermentation, 500 g/L glucose was fed when the glucose concentration fell below 10 g/L.

### Analytical technique

For the cell growth studies, OD_600_ was measured at a wavelength of 600 nm with a UV-1,800 spectrophotometer (Shimadzu, Kyoto, Japan), which has a linear correlation with the cell dry weight DCW (*Y* = 0.3628*x*, *Y* stands for the DCW, and *x* stands for the OD_600_) [16].

Fermentation broth was taken and centrifuged for 2 min at 13,000 rpm to remove cell debris, and subsequently filtered using a 0.22 μm filter, in preparation for substrate and metabolite analysis. A high-performance liquid chromatography system equipped with an Aminex HPX-87H column (BioRad, Inc., Hercules, CA) and a refractive index detector was used for analysis of glucose and succinic acid. 5 mM H_2_SO_4_ was used as the mobile phase with flow rate 0.6 mL/min at 65 °C.

### Phylogenetic analysis

Full-length amino acid sequences of C4-dicarboxylic acid transporters were obtained from GenBank and aligned by Clustal W. Amino acid sequence homology analysis was performed with BLASTp. The phylogenetic tree was constructed using the neighbor-joining method in MEGA software version 6.0.

## Supplementary Information

**Additional file 1: Table S1.** Strains used in this study. **Table S2.** Plasmids used in this study. **Table S3.** Primers used in this study. **Table S4.** Encoding sequences of C4-dicarboxylic acid transporters from different species. **Figure S1.** CRISPR interference (CRISPRi) system in *Y. lipolytica*. (a) Episomal vector pCRISPRi for the simultaneous expression of dCas9 and sgRNA; (b) functional verification of CRISPRi using *hrGFP* as the reporter gene. 4 different sgRNA targeting sites were designed in the front part of the *hrGFP* gene, its relative expression level could be as low as 27.6% of the control. **Figure S2.** Screening of transformants with overexpressed *YlScs2* (a) and *TbFrd* (b) through NHEJ-mediated random genome integration for SA production. **Figure S3.** Comparison of the SA titer and yield between different *Y. lipolytica* engineered strains after 72 h fermentation in shaking flasks.

## Data Availability

All data generated or analyzed during this study are included in this published article and its supplementary materials.

## Abbreviations

SA: Succinic acid
bio-SA: Biobased SA
MCs: Mitochondrial carriers
TCA cycle: Tricarboxylic acid cycle
NHEJ: Non-homologous end-joining
CRISPR: Clustered Regularly Interspaced Short Palindromic Repeats
CRISPRi: CRISPR interference
PCR: Polymerase chain reaction
HPLC: High performance liquid chromatography
OD_600_: Optical density at 600 nm
DCW: Cell dry weight
