# Supplementary material for: Engineering of Yarrowia lipolytica transporters for high-efficient production of biobased succinic acid from glucose
Source: Biotechnol Biofuels. 2021 Jun 27;14:145. doi: 10.1186/s13068-021-01996-w (PMC8237505; doi:10.1186/s13068-021-01996-w)
Supplement: Supplementary file 1 — Additional file 1: Table S1. Strains used in this study. Table S2. Plasmids used in this study. Table S3. Primers used in this study. Table S4. Encoding sequences of C4-dicarboxylic acid transporters from different species. Figure S1. CRISPR interference (CRISPRi) system in Y. lipolytica. (a) Episomal vector pCRISPRi for the simultaneous expression of dCas9 and sgRNA; (b) functional verification of CRISPRi using hrGFP as the reporter gene. 4 different sgRNA targeting sites were designed in the front part of the hrGFP gene, its relative expression level could be as low as 27.6% of the control. Figure S2. Screening of transformants with overexpressed YlScs2 (a) and TbFrd (b) through NHEJ-mediated random genome integration for SA production. Figure S3. Comparison of the SA titer and yield between different Y. lipolytica engineered strains after 72 h fermentation in shaking flasks. [file 13068_2021_1996_MOESM1_ESM.docx]

**Engineering of *Yarrowia lipolytica* transporters for high-efficient production of** **biobased succinic acid** **from glucose**

Zhennan Jiang ^a†^, Zhiyong Cui ^a†^, Ziwei Zhu ^a^, Yinghang Liu ^a^, Jin Hou ^a^*, Qingsheng Qi ^a, b^*.

^a^ State Key Laboratory of Microbial Technology, Shandong University, Qingdao, 266237, P. R. China

^b^ CAS Key Lab of Biobased Materials, Qingdao Institute of Bioenergy and Bioprocess Technology, Chinese Academy of Sciences, Qingdao, 266101, P. R. China

^†^ These authors contributed equally to this work.

* Corresponding author:

Qingsheng Qi

Mailing address: State Key Laboratory of Microbial Technology, Shandong University, Qingdao 266237, P. R. China; CAS Key Lab of Biobased Materials, Qingdao Institute of Bioenergy and Bioprocess Technology, Chinese Academy of Sciences, Qingdao 266101, P. R. China

Email: [qiqingsheng@sdu.edu.cn](mailto:qiqingsheng@sdu.edu.cn)

Jin Hou

Mailing address: State Key Laboratory of Microbial Technology, Shandong University, Qingdao 266237, P. R. China

Email: houjin@sdu.edu.cn

**Table S1** Strains used in this study.

| Name | Description | References |
| --- | --- | --- |
| PGC62 | MatA, xpr2-322, axp-2, leu2-270, ura3-302, *ΔSdh5::loxP*, *ΔAch1::loxP*, *ScPck* | ([Cui et al., 2017](#_ENREF_1)) |
| PGC62-SpMae | Overexpression of *SpMae1* in PGC62 strain | This study |
| PGC62-ScMae | Overexpression of *ScMae1* in PGC62 strain | This study |
| PGC62-CtMae | Overexpression of *CtMae1* in PGC62 strain | This study |
| PGC62-YlMae | Overexpression of *YlMae1* in PGC62 strain | This study |
| PGC62-AfMae | Overexpression of *AfMae1* in PGC62 strain | This study |
| PGC62-CcMae | Overexpression of *CcMae1* in PGC62 strain | This study |
| PGC62-VpMae | Overexpression of *VpMae1* in PGC62 strain | This study |
| PGC62-MsMae | Overexpression of *MsMae1* in PGC62 strain | This study |
| PGC62-RaMae | Overexpression of *RaMae1* in PGC62 strain | This study |
| PGC62-YlCtp1 | Overexpression of *YlCtp1* in PGC62 strain | This study |
| PGC62-YlDic | Overexpression of *YlDic1* in PGC62 strain | This study |
| PGC62-YlDici | Inhibition of *YlDic1* in PGC62 strain through CRISPRi method | This study |
| PGC62-YlOdc | Overexpression of *YlOdc1* in PGC62 strain | This study |
| PGC62-YlAcr | Overexpression of *YlAcr1* in *PGC62* strain | This study |
| PGC62-YlCtp2 | Overexpression of *YlCtp2* in *PGC62* strain | This study |
| PGC62-Frd | Overexpression of *TbFrd* in PGC62 strain | This study |
| PGC62-Scs | Overexpression of *YlScs2* in PGC62 strain | This study |
| PGC62-YMI | Overexpression of *YlIcl, YlMls and YlYhm2* in PGC62 strain | This study |
| PGC62-FS | Overexpression of *TbFrd and YlScs2* in PGC62 strain | This study |
| PGC62-FY | Overexpression of *TbFrd, YlScs2, YlIcl, YlMls and YlYhm2* in PGC62 strain | This study |
| PGC62-SY | Overexpression of *YlScs2, YlIcl, YlMls and YlYhm2* in PGC62 strain | This study |
| PGC62-SYF | Overexpression of *TbFrd, YlScs2, YlIcl, YlMls and YlYhm2* in PGC62 strain | This study |

**Table S2** Plasmids used in this study.

| Name | Description | Source |
| --- | --- | --- |
| pKi-hyg | Integrative vector with the *HYG* marker and pUAS1B-TEF promoter | ([Cui et al., 2019](#_ENREF_2)) |
| pKi-hyg-SpMae | Integrative vector with the *HYG* marker and *SpMae* expression cassette | This study |
| pKi-hyg-CsMae | Integrative vector with the *HYG* marker and *CsMae* expression cassette | This study |
| pKi-hyg-CtMae | Integrative vector with the *HYG* marker and *CtMae* expression cassette | This study |
| pKi-hyg-YlMae | Integrative vector with the *HYG* marker and *YlMae* expression cassette | This study |
| pKi-hyg-AfMae | Integrative vector with the *HYG* marker and *AfMae* expression cassette | This study |
| pKi-hyg-CcMae | Integrative vector with the *HYG* marker and *CcMae* expression cassette | This study |
| pKi-hyg-VpMae | Integrative vector with the *HYG* marker and *VpMae* expression cassette | This study |
| pKi-hyg-MsMae | Integrative vector with the *HYG* marker and *MsMae* expression cassette | This study |
| pKi-hyg-RaMae | Integrative vector with the *HYG* marker and *RaMae* expression cassette | This study |
| pKi-1 | Integrative vector with the *LEU2* marker and pUAS1B-TEF promoter | ([Cui et al., 2019](#_ENREF_2)) |
| pKi-YlCtp1 | Integrative vector with the *LEU2* marker and *YlCtp1* expression cassette | This study |
| pKi-YlDic | Integrative vector with the *LEU2* marker and *YlDic1* expression cassette | This study |
| pKi-YlOdc | Integrative vector with the *LEU2* marker and *YlOdc1* expression cassette | This study |
| pKi-YlAcr | Integrative vector with the *LEU2* marker and *YlAcr1* expression cassette | This study |
| pKi-YlCtp2 | Integrative vector with the *LEU2* marker and *YlCtp2* expression cassette | This study |
| pKi-Frd | Integrative vector with the *LEU2* marker and *TbFrd* expression cassette | This study |
| 113-GPD-TEF | Integrative vector with the *URA3* marker, GPD promoter and TEF promoter | ([Cui et al., 2019](#_ENREF_2)) |
| 113-Scs | Integrative vector with the *URA3* marker and *YlScs2* expression cassette | This study |
| 113-YMI | Integrative vector with the *URA3* marker, *YlIcl*, *YlMls* and *YlYhm2* expression cassette | This study |
| pCAS1yl-trp | Plasmid for CRISPR/Cas9, sgRNA targeted to *TRP1* | ([Gao et al., 2016](#_ENREF_3)) |
| pCAS1yl-Dic | Plasmid for CRISPRi, sgRNA targeted to *YlDic1* | This study |
| YLEP-leu | Episomal vector with the *LEU2* marker and pUAS1B-TEF promoter | ([Cui et al., 2019](#_ENREF_2)) |
| YLEP-hrGFP | Episomal vector with the *LEU2* marker and hrGFP expression cassette | This study |

**Table S3** Primers used in this study.

| Name | Sequence | Use |
| --- | --- | --- |
| SpMae-F | TATAAGAATCATTCAAAGGTTATGGGCGAGCTGAAGGAAATC | Amplifying *SpMae1* |
| SpMae-R | CGTGACATAACTAATTACATGATTTTACACAGACTCGTGCTCAGAAGAT | Amplifying *SpMae1* |
| ScMae-F | TCTGAGTATAAGAATCATTCAAAGGTTATGGCCGACGTGAAGGG | Amplifying *ScMae1* |
| ScMae -R | AGCGTGACATAACTAATTACATGATTTTATCGGTGGTGCACGGTAGAA | Amplifying *ScMae1* |
| CtMae-F | TATAAGAATCATTCAAAGGTTATGGACCGACGAGATCCCAT | Amplifying *CtMae1* |
| CtMae-R | TTAGTCCAGCACGTCCTCGTCTTAGTGGTCGTTGAAGTCCTCATCT | Amplifying *CtMae1* |
| YlMae-F | TGAGTATAAGAATCATTCAAAGGTTATGACCACACCTCAACCACG | Amplifying *YlMae1* |
| YlMae-R | CGTGACATAACTAATTACATGATTTTACTGCTCCAAAGGATCAGTTTCG | Amplifying *YlMae1* |
| AfMae-F | CGTGACATAACTAATTACATGATTTTACTGCTCCAAAGGATCAGTTTCG | Amplifying *AfMae1* |
| AfMae-R | CGTGACATAACTAATTACATGATTTTAGTCCAGCACGTCCTCGTC | Amplifying *AfMae1* |
| CcMae-F | TGAGTATAAGAATCATTCAAAGGTTATGFTTCNAACDGACHCACDGACHCAC | Amplifying *CcMae1* |
| CcMae-R | AATCATGTAATTAGTTATGTCACGCACGAGGACGTGCTGGACTAA | Amplifying *CcMae1* |
| VpMae-F | TGAGTATAAGAATCATTCAAAGGTTATGACCTTCCGATCTGCCGT | Amplifying *VpMae1* |
| VpMae-R | AATCATGTAATTAGTTATGTCACGCTTATCGTC | Amplifying *VpMae1* |
|  | GAGCGCCCAGG |  |
| MsMae-F | TGAGTATAAGAATCATTCAAAGGTTATGCACCGAGATCGAGATCAGG | Amplifying *VpMae1* |
| MsMae-R | AATCATGTAATTAGTTATGTCACGCTTAGCCGATGATTCGGTGGGT | Amplifying *VpMae1* |
| RaMae-F | TGAGTATAAGAATCATTCAAAGGTTATGTCTTACCAGGCTGGCCC | Amplifying *RaMae1* |
| RaMae-R | AATCATGTAATTAGTTATGTCACGCTTAGTGGGCGTCCTCATCTCG | Amplifying *RaMae1* |
| 323-F | CCTTCTGAGTATAAGAATCATTCAAAGGTTATGGTTTCATCAGATACCAAGAAGGC | Amplifying *YlCtp1* |
| 323-R | TAAGCGTGACATAACTAATTACATGATTCTAAAGAATCTCCATGATCTTCTCATAGATG | Amplifying *YlCtp1* |
| 344-F | CCTTCTGAGTATAAGAATCATTCAAAGGTTATGTCATCTTTACAAAAACACTTACCCAT | Amplifying *YlDic1* |
| 344-R | TGTAAGCGTGACATAACTAATTACATGATTCTAATGTCGCATTCCAATCTTGTAGAACT | Amplifying *YlDic1* |
| 629-F | CCTTCTGAGTATAAGAATCATTCAAAGGTTATGTCTGACCAGAAGCCCCT | Amplifying *YlOdc1* |
| 629-R | TGTAAGCGTGACATAACTAATTACATGATTTTATTTCTTACCGTCGTGGATTCCTCG | Amplifying *YlOdc1* |
| 672-F | CCTTCTGAGTATAAGAATCATTCAAAGGTTATGGCTGCTGACGGAAAGAAG | Amplifying *YlAcr1* |
| 672-R | TGTAAGCGTGACATAACTAATTACATGATTTTACTCCTCAAACTGGGCAGCAAA | Amplifying *YlAcr1* |
| 966-F | CCTTCTGAGTATAAGAATCATTCAAAGGTTATGAAGACGTCGCTCCAGC | Amplifying *YlCtp2* |
| 966-R | TGTAAGCGTGACATAACTAATTACATGATTCTA TTCCTCTCCAGGGGGGAAAG | Amplifying *YlCtp2* |
| Frd-F | TGAGTATAAGAATCATTCAAAGGTTATGGTGGACGGTCGATCTTC | Amplifying *TbFrd* |
| Frd-R | AGCGTGACATAACTAATTACATGATTTTAGGAGCCAGAGGGCTCG | Amplifying *TbFrd* |
| Scs-F | TTTTGCAGTACTAACCGCAGATTTATGTTTTCGCGAATTGCTGC | Amplifying *YlScs2* |
| Scs-R | TGACATAACTAATTACATGAATTTTTAAAGAGGCAGCTCAAAGGAGAC | Amplifying *YlScs2* |
| Icl-F | AATTAAACACACATCAACAGATGTCCGAACAGCAGCGAT | Amplifying *YlIcl* |
| Icl-R | GGGGACAGGCCATGGAGGTACGTTAAAGCTTGGACTTGAACTGGTCCTC | Amplifying *YlIcl* |
| Mls-F | TGCAGTACTAACCGCAGATTTATGACTACTGTTCCTTCTAGCAACACTG | Amplifying *YlMls* |
| Mls-R | ACATAACTAATTACATGAATTTTTAGAGCTTGGAAGAGCTGCCAA | Amplifying *YlMls* |
| Yhm-F | ACACAAGACATATCTACAGCATTTATGGGTGCTGCTAACCTCAAC | Amplifying *YlYhm2* |
| Yhm-R | ATTTTGCTAAACAAACTGCATTTCTAGTGCTTACCAACAGGTCGC | Amplifying *YlYhm2* |
| CRISPRi-F | CTCGAGGGGGGGCCCGGTACTCTGTACAGAAAAAAAAGAA | Construction of pCRISPRi |
| CRISPRi-F1 | GCCATCGTGCCCCAGTCTTTTCT | Construction of pCRISPRi |
| CRISPRi-R1 | AAGACTGGGGCACGATGGCATCCACGTCGTAGTC | Construction of |
|  | GGAGA | pCRISPRi |
| CRISPRi-F2 | GCCATCGGCACAAACAGCGTCGG | Construction of pCRISPRi |
| CRISPRi-R2 | CGCTGTTTGTGCCGATGGCGAGCCCAATGGAGTA | Construction of |
|  | CTTCT | pCRISPRi |
| CRISPRi-R | ACTAAAGGGAACAAAAGCTGGTACGTTTAAACAGAGACCG | Construction of pCRISPRi |
| sgRNA-GFP1-F | CCTGCAGGCCGGTGTTCTTCGTTTTAGAGCTAGAAATAGC | Construction of pCRISPRi-GFP1 |
| sgRNA-GFP1-R | GAAGAACACCGGCCTGCAGGGACGAGCTTACTCGTTTCGT | Construction of pCRISPRi-GFP1 |
| sgRNA- GFP2-F | AGCCCTCCATGGTGAACACGGTTTTAGAGCTAG AAATAGC | Construction of pCRISPRi-GFP2 |
| sgRNA-GFP2-R | CGTGTTCACCATGGAGGGCTGACGAGCTTACTCGTTTCGT | Construction of pCRISPRi-GFP2 |
| sgRNA-GFP3-F | GAAGAAGTCGCTGATGTCCTGTTTTAGAGCTAGAAATAGC | Construction of pCRISPRi-GFP3 |
| sgRNA-GFP3-R | AGGACATCAGCGACTTCTTCGACGAGCTTACTCGTTTCGT | Construction of pCRISPRi-GFP3 |
| sgRNA-GFP4-F | TGCGGCCCTTGTACTCCACGGTTTTAGAGCTAGAAATAGC | Construction of pCRISPRi-GFP4 |
| sgRNA-GFP4-R | CGTGGAGTACAAGGGCCGCAGACGAGCTTACTCGTTTCGT | Construction of pCRISPRi-GFP4 |
| sgRNA-Dic-F | TGGGTAAGTGTTTTTGTAAAGACGAGCTTACTCGTTTCG | Construction of pCRISPRi-Dic |
| sgRNA-Dic-R | TTTACAAAAACACTTACCCAGTTTTAGAGCTAGAAATAG | Construction of pCRISPRi-Dic |

**Table S4** Encoding sequences of C4-dicarboxylic acid transporters from different species.

| Name | Sequence |
| --- | --- |
| *SpMae1* | ATGGGCGAGCTGAAGGAAATCCTGAAGCAGCGATACCACGAGCTGCTGGACTGGAACGTGAAGGCCCCTCACGTGCCCCTGTCTCAGCGACTGAAGCACTTCACCTGGTCCTGGTTCGCCTGCACCATGGCCACCGGCGGAGTGGGCCTGATTATCGGCTCTTTCCCCTTCCGATTCTACGGCCTGAACACCATCGGCAAGATCGTGTATATCCTGCAGATCTTCCTGTTCTCTCTGTTCGGCTCTTGCATGCTGTTCCGATTCATCAAGTACCCCTCTACCATCAAGGACTCTTGGAACCACCACCTCGAGAAGCTGTTCATTGCCACCTGTCTGCTGTCTATCTCTACCTTCATCGACATGCTGGCCATCTACGCTTACCCCGACACCGGTGAGTGGATGGTGTGGGTGATCCGAATCCTGTACTACATCTACGTGGCCGTGTCTTTCATCTACTGCGTGATGGCCTTCTTCACCATCTTCAACAACCACGTGTACACCATCGAGACTGCTTCTCCCGCCTGGATTCTGCCTATCTTCCCTCCTATGATCTGCGGCGTGATCGCTGGCGCCGTGAACTCTACCCAGCCTGCTCACCAGCTGAAGAACATGGTGATCTTCGGCATCCTGTTCCAAGGCCTCGGCTTCTGGGTGTACCTGCTGCTGTTCGCCGTCAACGTGCTGCGATTCTTCACCGTCGGCCTGGCTAAGCCCCAGGACCGACCTGGCATGTTCATGTTCGTGGGCCCTCCAGCCTTCTCTGGACTGGCCCTGATCAACATTGCCCGAGGCGCCATGGGCTCTCGACCCTACATTTTCGTGGGCGCCAACTCTTCTGAGTACCTGGGCTTCGTGTCCACCTTCATGGCCATCTTCATCTGGGGCCTCGCCGCCTGGTGCTACTGCCTGGCTATGGTGTCTTTCCTGGCCGGCTTCTTCACTCGAGCCCCTCTGAAGTTCGCCTGTGGCTGGTTCGCTTTCATCTTCCCCAACGTGGGATTCGTGAACTGCACCATTGAGATCGGAAAGATGATCGACTCTAAGGCCTTCCAGATGTTCGGCCACATCATCGGAGTGATCCTGTGCATCCAGTGGATCCTGCTGATGTACCTGATGGTGCGAGCCTTCCTGGTGAACGACCTGTGCTACCCCGGCAAGGACGAGGACGCTCACCCTCCACCTAAGCCTAACACCGGCGTGCTGAACCCCACCTTTCCACCTGAGAAGGCTCCCGCCTCTCTCGAGAAGGTTGACACCCACGTGACCTCTACCGGCGGTGAGTCTGACCCTCCATCTTCTGAGCACGAGTCTGTGTAA |
| *ScMae1* | ATGGCCGACGTGAAGGGCATGCTGCGACAGCGATACCACGAGCTGCTGGACTGGCAGGTCAAGTCTCCCCACGTGCCTCTGTCTCAGCGAATCAAGCACTTCACCTGGTCCTGGTTCGCCTGCACCATGGCCACCGGCGGCATCGGCCTGGTGATCGGCACCTTTCCATTCCGATTCCGAGGCCTGGACACCATCGGCAAGATCGTGTATATCTTCGACATCTTCCTGCTGGCCCTGTTCTCTTGCTGCATGATCGTGCGATTCGTGAAGTACCCCGGCACCTTCCTCGGCTCTTGGAAGCACTTTCAAGAGAAGTTCTTCATTGCCACCTGTCTGCTGTCTTTCTCTTCTTTCATCGACATGTTCGCCGTGTACGCTATGCCCAACACCGGCGAGTGGATGATCTGGGTGATCCGAATCTTCTTCTACATCTACCTGGCCGTGACCTTCCTGTACGGAACCTTCGCCTACTACACCATCTTCCGAGATCACGTGTACACTCTCGAGGGCGCTGCTCCCACCTGGGTGCTGCCTATCTTCCCCTGTATGATCACCGGCGTGGTGTCTGGCTCTGTGGTGTCCTCTCAGCCCTCTGCTCAGCTGAAGAACATGGTGATCCTGGGCATCATGTTCCAAGGCCTCGGCTTCTGGGTGTACCTGCTGGTGTACTCTATCCTGATTCTGCGATTCTTCACCATCGGATTCGCTAAGCCCGCCGAGCGACCCGGCATGTTCATCCTGGTGGGACCCGCCGGATTCACCGGACTGGCCCTGATCAACATGGCCCGAGGCGCCATTGCCACTCGACCCAACATCTTCGCCTCTGCCAACTCTTCTGAGTACTTCGCCTTCACCTCTACCTTTCTGGCCCTCTTCATCTGGGGCCTCGGCGCCTGGACCTACTGCTTCGCCATGGTGTCTTTCGTGGCCGGACTGTTCTCTCACCAGCCTATGAAGTTCTCTAACACCTGGTTTGCCATGATCTTCCCCAACGTGGGCTTCGTGCTGTGCACCGTGCGAATCGGCCAGATGATCAACTCTAAGGCTTTCACCCTGTTCGGCCACATCATCTGCGTGATCCTGTGTATTATGTGGCTGATCCTGATGTATATGATGATCCGAGCCTTCCTGGTGAACGACCTGATGTACCCCGGAAAGGACGAGGACTCTAAGTCTCCCGCCGAGTCTCGACCCATTGCCGTCGAGCCCGAGAAGTTCGGCATCCCCAAGTCTCAGCCTGAGAACTCTCTGGACGTCGAGAAGGCTGACAACCCTCTGGACTCCGCCAACCACGGCGCCGACCACGACCGAGACTCCTCTTCTACCGTGCACCACCGATAA |
| *CtMae1* | ATGGACCGACGAGATCCCATGAACTCTGCCTGGACCGAGTCTGAGGTGTCTACCCGAAAGAACTCCGCCGAGTGGCAGACCTCTCGACCCAACTCTCCCAAGCTGGCTCCCCTGGACATGCAGTTCGACAAGGGCAAGGCCAACGAGAAGGACGGCTACGCCACCTCTATCACCCCTATCATCGAGAAGGGCGCTGCTCCCGCTCACGACCACGGCCACGGACACATCGACATCAACGACCCCAACCGACCTCGAATGGCCTTCAAGGCCCGACTGCACCACTTCACCTGGGCCTGGTACACCCTGACCATGTCTACCGGCGGACTGTCTCTGCTGATCTTCGCTCAGCCCCATCAGTTCCCCGGCCTGCGACAGATCGGCACCGTGGTGTACGTGGTGAACATCATCCTGTTCGTGCTGGTGTGCTCTGCTATGCTGGCCCGATTCTTTCTGTACCCCGGCGACATGAAGAAGTCTCTGACCCACGAGCGAGAGGGATTCTTCTTCCCTACCTTCTTCCTGTCTATCGCTACCCTGATCACCTCTACCAACCGATACGCTATCCCCGAGCACGACGAGACTCTGGTGTGGGCCATCCAGGTGGCTTTCTGGGGCTACCTGATCGTGACCCTGATGCTGGCTATCGGCCAGTACTCTTTCGTGTTCGCCAAGCACAACTTCGGCCTGCAGACCATGATGCCTACCTGGATCCTGCCTATCTTCCCCATCATGCTGACCGGCACCATTGCCTCTGTGATCGCCGACACTCAGCCCGAGATCGCCGCTGTGCCCATCGTGGTGGCCGGCCTGACCTGCCAAGGCCTGGGACTGTCTGTGGCCGTCCTGATGTACGCCCACATGGTGGGCCGACTGATGTCTGCCGGACTGCCCAACCGAGAGCACCGACCTGGCCTGTACATGAACGTGGGCCCTCCAGCCTTCACCGCTCTGGCCCTGATCGGCATGGCCAACGGTCTGCCCAACAACTTGGACCCCGACCGAGATGGCATCATCATCGACGCCGGCATCATCCGAACCATTGCTCTGATGTCCGCCATCTTCCTGTGGGCTCTCGCCGCCTGGTGGTTCGGAATCGCCACTATCGCCGTGATCTCTTCTCCTCCTGTGTACTTCCACCTCGGCTGGTGGGCCATGGTGTTCCCCAACACCGGCTTCACCCTGGCCACCATCTCTATCGGCAACCAGCTGGGCAACGAGGCCGTGCTGTGGTTCGCCACCGGCATGTCTCTGTGCCTGCTGGGCGCCTACTTCTACGTGCTGTACCACCACGTGCGAGCCGTGATCATCCAGGACATCATGTACACCATGCGAGATGAGGACTTCAACGACCACTAA |
| *YlMae1* | ATGACCACACCTCAACCACGAGCAATGACCGACCTCGAAATGGCTCCTGCTCCAACAACGTCGTCCCAGGAGATCCAGGGAAAGGGCTCCAACCACATCGCATCGTCGCCAGACGCAACCAACGACAAGAGCCGGCCAGAAATGTCCTATCTGATGCTGCGTATCAAGTACTTTAGCCCCGCGTGGTTTGCGTCGGTCATGGGCGTGGGCGTGTCCGGAGGCATTCTGTACACGTATCCATTCCACGCGCGATGGCTGGAGATTCTGGGCGAGGTCATCTGGGCCATTTCTTTGGGCATGCTGGGAGTCTTCTCCGTCCTGTTTTGCGCCCGGTTTGTGCTCTTCCCCAAGCAGTTTATGAAAATGCTCAAACACCCCGGCCAATCGACCTTTCTGGGCTGCATCCCCATGGCCTTCTGCACCACCATCAACATGATCCACAACATCTGGGGTCAGGACGGATGGCTTGCATGCTACATTCTCTGGTGGTTCAACGTGGTCATGTCTCTGGCCACTTGCTGGGGAGTCACCGTGTCCATGTTCTACCTCCACAAACGAACCGCCACCATGCTCAACGCCACCATGATTCTTCCCATCGTGTCTGTGGTCGTCTGTGCCGCTACAGGTACCCTCTTCTGGGATAACGTCCCCCACCACCTCCAGGGCCTCCAGCTCGTCGTCTGCATCATGCTCTGGGCCAACGGTCAGTGTCTCGCCTTTGGGTTTGTCACCGTGTACCTCTGGCGTCTATTGTCCATCGGCATGGTCCCCCCGGCTCTTGTCATCTCCAACTTTTTGCCCGTGGGCCCTCTGGGCCAAGGCTCATTCGGTATTCTGCTCATGTCCAGTGCCGCCCAAAAGTACCTGCTCAGACAGTTCCCCAACGACGCTCTGGATGCAGAGGTGATGGGCCAGCTGACCAACACCGCTAGCCGGGTAGTTTTCACGCAGTTCTACGAGTTCCTCGGCATCTTCATCGCCCTCTTTCTCCAGGGCTTTGCCTTCTTCTGGCTCTTTGTGGCCTTCACGTGCTTGGCCTACACTCCTCCCAAGCAGTTTGCCATTGGTTGGTGGGGCCTGACTTTCCCGCTGGGCACCTTTGCCCTGGGAACCGCCCGAATGGGCGTCGAGCTGGATTCTCTTGCATTCAGAATCATTAGCGGCATTTCGGGCGTCATGGTCTGTCTCTTTACGCTGATTTGTGTTCTCGGCTCCATCCGTGACGGCATTATTGGCAACAAAATCTTCCTGGCCTCCCAGGACGAAACTGATCCTTTGGAGCAGTAA |
| *AfMae1* | ATGTTCAACGACCACGACCACGTGCCTCCAACCTCTTCTCAGTCTGACTCTGGCTTCTTCGAGCAAGAGATGAAGAAGTCTCCCCGACTGTCTCTGCGAGAGCGACTGCGACACTTCACCTGGGCCTGGTACACCCTGACCATGTCTACCGGCGGACTGGCCCTGCTGATCGCCTCTCAGCCCTACACCTTCAACGGCATGAAGGGCATCGGCATGGTGGTGTACATCCTGAACCTGCTGCTGTTCGCCCTGGTGTGCTCTCTGATGGTGCTGCGATTCGTGCTGCACGGCGGCTTTCTGGACTCTCTGCGACATCCCCGAGAGGGCCTGTTCTTCCCCACCTTCTGGCTGTCTATCGCCACCATCATCACCGGCCTGCACCGATACTTCGGCTCTGACGACCTGGAATCTTACCTGATCGCCCTGGAAGTGCTGTTCTGGGTGTACTGCTCTTGCACCCTGGCCACCGCCGTGATCCAGTACTCTTTCCTGTTCGCCGCTCACTCTTACGGCCTGCAGACCATGATGCCCTCTTGGATTCTGCCTGCTTTCCCCATCATGCTGTCTGGCACCATTGCCTCTGTGATCTCTGAGTCTCAGCCCGCTCGATCTGCTATCCCCATTATCACCGCTGGCGTGACCTTCCAAGGCCTGGGCTTCTCTATCTCTTTCATCATGTACGCCCACTACATCGGCCGACTGATGCAGTCTGGACTGCCCTGCCGAGAGCACCGACCTGCCATGTTCATCTGCGTGGGCCCTCCATCTTTCACCGCTCTGGCCCTGGTCGGCATGGCCAAGGGACTGCCCGACGAGTTCAAGATCATCAAGGACGCCCACGTCGAGGACGCCCGAATCCTGGAACTGATGGCTATTATCGTGGGCGTGTTCCTGTGGGCCCTGTCTCTGTGGTTCTTCTTCATTGCCTTCGTGGCCGTGGTCCGATGTCGACCCACCGCCTTCCACCTGTCTTGGTGGGCCATGGTGTTCCCCAACACCGGCTTCACCCTGGCTACCATCACTCTGGGACGAGCCCTGGGATCTCCCGGCGTGCTCGGCGTGGGATCTGCCATGTCTGTGGGCGTCGTGTGCATGTGGGTGTTCGTGTTCGTCTACCACATCCGAGCCGTGATTCGACAGGACATTATGTACCCCGGCAAGGACGAGGACGTGCTGGACTAA |
| *CcMae1* | ATGTCTCTGGAATCCCGAGAGGGACGAAACCCTCGATTCCAAGAGACTGGCCCCGAGAAGCACAACACCGACAACCGAAAGGTGTCTCTGCGACAGCGAATCCACCACTTCACCTGGGCCTGGTACACCCTGACCATGTCTACCGGCGGCATGGCCCTGCTGCTGTCTGTGACCCCTCATCGATTCCCCGGCCTGGACACCATCGGCGCCATCGTGTACATCTTCGACCTGGTGCTGTTCACCTGTATCTCTATGGCCATCACCGCTCGATTCGTGATGTTCAAGGGCACCCTGTTCCAGTCTCTGACTCACCCCACCGAGTCTCTGTTCTTCGGCACCATGCTGCTCTCCCTGGCCACCATCATCTGCGGCATGCAGAAGTACGGCGAGCCCAACGTCGGCGGCTGGCTGGTGGTCGTGCTGCGAATCGTGTTCTGGATCTACTGCGCTACTACCTTCCTGGTGGCCTCTTTTCAGTACTGGCGACTGTTCGACGGCCAGCACATGCCCATCCTGTCTATGTCTCCCGCCTGGATTCTGCCTATCTTCCCCGTGATGCTGTCTGGCACCATTGCCAACGTGATCTCGCCCTCTCAGCCCGACATTCACGCCCGAGACATCATCGTGGCCGGCGTGACCTTCCAAGGCCTGGGAATGATGGTGTCTTTCCTGATGTACTCTTCTTTCATCGGCCGACTGATGCAGTCTGGACTGCCCGAGCCTAACTCTCGACCCGGCATGTTCATTGCCGTGGGACCTCCATCTTTCACCGCTCTGGCCCTGATCGGAATGGCCAACTCTTCTTCTGTGTTCACCGCCTCTATCATCGAGACTGCCGTGGTGCCCGCTGACGTGCTGAAGATTATGGCCCTGTTCCTGGCCATCTTCCTGTGGTCCCTGTCTCTGTGGTTCTTCTCTATCTCTCTGATCTCTGTCCTGGCCGTGGCCGCCAAGATGTCTTTCCACCTGTCTTGGTGGTCGCTGGTGTTCCCCAACATCGGCTTCACCATTGCTACCATCGAGATCGGAACCCTGCTGAAGTCTGAGGGCATCCTGTGGCTGGGCTCTGTGATGACCGTGATGATCGTCGCCATCTGGCTGTTCGTGATCGTGGCCCACGTGCACGCCGTGACCACCTCTAAGATCATGTACCCCGGCAAGGACGAGGACAAGGAAGAGGAATACGCCGGCATCCACCAAGAGTAA |
| *VpMae1* | ATGACCTTCCGATCTGCCGTCCAGGTGTTCAAGGAAGAACGACTGCCCCACTTCACCTGGGCCTGGTACACCCTGCCTATGTCTACCGGCGGACTGTCTCTGCTGCTGTCTGTGACCCCTAACCGATTTCCTGGCCTGACCACCATCGGCGTGGTGCTGTACATCTGCAACATCGTGATCTTTCTGTCTATCTCTTCTATCATGATCTGGCGATACTCTTCTTGCAACGTGACCTTCAAGGATACCCTGATGCACCCTCTGGAATCTCTGTTTATCCCTACCTTCTTCCTGGCCACCGCCACCATCATCAACTGCATGTGCGCCTACGCTGTGCCCAAGACCGGCGAGTGGATGGTGGTGCTGCTGCGAGTGATCTTCTGGATCTACACCGCTACCACCTTTTGCCAGTCTGTGATCCAGTACTTCATCCTGTTCAACAACCGACAGCACCCCATTCAGAACATGACCACCGCCTGGGTGCTGCCTGCTTTCCCCTGCATGCTGGTGGGCACCGTGGCCTCTGCCGTGGTCAAGACCCAGCCTCGACAGCACGCCTTCAACATGGCCCTGGCCGGCACCACCTGTCAAGGCCTGGGCTTCCTGATCTCTTGCTTCATGTACTCTATCCTGATCCGACGACTGATGCAGTTCGGACTGCCCGAGATCAAGGCTCGACCCGGCATGTTCATTACCGTGGGACCTCCATCTTTCACCTGTCTGGCCCTGCTGGGACTCGCCCAGGACGCCTCTTACTTCCTGCCTGAGAAGTTTCTGACCCTCAAGACCACCGACGTGTCTGAGATCCTGCTGATCATGGGCACCACCGGCTCTGTGCTGCTGTGGTCTGTGTCTGCCTGGTTCTGGGCCCTGACCATGGTGTCTATCCTGGCCGGACTGATTCAAGAGCCCCAAGAGATGCGATTCATCCTCGGATGGTGGGCCTTCGTGTTCCCCAACGTGGGCTTCACCATTGCCACCATCGAGCTGTCTAAGGTGTTCGACTCTTCTGCCCTCGGCTGGCTGGCCTCTGGCATGACCATCCTGATCTTCGCCGCCTGGCTGTTCGTGCTGACCTTTAACATCGAGGCCGTGGTGAAGGGCCGAATCATGGGAAAGGGCCTCGACGAGGACCGAGAGATCCGATTCGACTCTCACCTGGAAGCCCGAGTCGAGGACCTGGAATGCAAGGTCGAGCTGCCCCGACACCTGTCCGGCTTCTCTAAGGACTACCGAATGGGCGAGAAGTTCGACGAAGAGGAAGAGGAACGAATCCGAGAGTTCCACCGAGGCCGAGGCATGTCTTGCGCCTCCGGAATGACCACTGTGGCTCCCACTCCTATGATCTCTCGAAAGCCCTCTGACGACGAGAACATGCCCTCTTCTACCCTGGGCGCTCGACGATAA |
| *MsMae1* | ATGCACCGAGATCGAGATCAGGTGTACGCCGTGCACAACTACGTGAACAAGGAACCCGCCGTGGTCAAGACCGCTCCTGTCGAGTCTAACACCGAGGTGAAGGTGGACTCTCCCAACCTGTCTGAGCCCCGAAACTTCTTCGACCGACTGGAACACTTCACCTGGGCCTGGTTCACCCTGCCTATGGCCACCGGCGGACTGGCCCTGCTGCTGAACTCTGGAACCCAGCCTCACTCTTTCGACGGCCTGAACACCATCGGCAAGGTGGTGTACATCTTCGACCTGGTGGTGTTCGTGACCCTGTGCTCTTGCATCACCTACCGATTCGTTCGATGGCCCCAGACCTTCCGAGAGTCTCTGACCCATCCTACCGAGTCTCTGTTCCTGGGACCTCTGTTTCTGTCCATGGCCACCATCATCTCTTGCATGGGCCGATACGGCGAGCCCGCCACCGGACCTTGGCTGATCTGGGTGCTGCGAATCCTGTTCTGGATGTACGTGGCCGTGACCTTCATCCTGGCCGTGTTTCAGTACGTGGTGCTGTTCACCTCTCCTAAGCTGAAGCTGATCGACATGACCCCTGCTTGGGACCTGCCTATCTTCCCCTTCATGCTGTCTGGCACCATTGCCTCTGTGACCGGCGGAATGCAGCCTCCTGAGCACGCTGTGCCCATCCTGGTGGCCGGCATCACCGCTCAAGGCCTGGGCTTCACCATTGCTACCCTGATGTCTGTGCTGTACGTGCGACGAATGATCGAGTTCGGCCTGCCTCCTCCTGCCGCTCGACCCGCCATGTTCATTGCCGTGGGACCTCCATCTTTCACCGCTCTGGCCCTGTTCGGCATGGCCAACAACTTTCCCCGAGACTACATCAACTACTTCTCTTACTACCTGCCTTTCGGCTTCTCTGGACCCGGCGAGGCCTTCTCTAACGAGCAGCAGCAGCTGATCGGCGTGCAGATCACCGTGACTCTGGCCATCTGGGTCGCCGTGTTCATCTGGTCTATGGGCGTGTGGTTCTTCTGCGTGGCCCTGATCTCTACCCTCATGGTGGCTCGACAGCTGAAGTTCAAGCTGAACTGGTGGGCCTTCGTGTTCCCCAACACCGGCCTGACCATTGCCACTATCACCCTGGCCAAGGCCTTCCGATCTGAGGGCTTCAAGTGGGTGGGCTCTATCATGTCTATCCTGATCGTGGCCACCTGGCTGTTCATCTTCGTGATGCACGTGCGAGCCATTTTCATGAAGCAGATTCTGTGGCCCGGCAAGGACGAGGACGTGTACGTGCAGCACGAGGACTACAAGGGCCGACGACGAGATCGAATCCAGCAGCGACGACTGTCTCGAAACATGTCCTCTGATCTGGACCCCGAGTGGCACGCTTCTACCCACCGAATCATCGGCTAA |
| *RaMae1* | ATGTCTTACCAGGCTGGCCCCACCTCTGTGGACGACCTGGTGAACTACAAGAAGGCCATCCTGCGGGACGACCGACGAGACACCCGAATCCGAGGCTCTATCGAGGCTACCCTTGAGTTCTTCAAGTCTAAGCAGTCTGACGCCCCTCCTCTGCCTGCCGGCCGACCTGCTTACCCCACCGACTCTTCCGGCCTGAACAACACCATGTCGTACCCTCAGATCTCTCGACCCACCATGGCTCCCCAGTACAACGACTACAACGATATGGAAAAGCGACCCTCTAACTTCAACGACTTCAAGTCCGAGAAGATGGACATCGAGGCCAAGCCTGCCGTGCACCACGACGACCACCACGGCATCTCTGCTGGACCTCACACTCTGAAGGAACGACTGAAGCACTTCACCTTCTCTTGGTACGCCTTCACCATGGCCACCGGCGGCACCGCTCTGACCCTGTCTGTGGTGCCCAACCGATTCTCTGGCCTGACCTCTCTGGGAACCTTCATGTTCCTGCTGAACATCTTCTTTTTCATCTGCGTGACCTCTGCCATGATCACCCGATTCATCATCTACCCCGAGATCTTCATCTCTTGCTTCACCAACTTCCACGAGGGCGTGTTCTCTGCCACCTTCTGGCTGACCTGCGCCACCATGATTACCAACACCGTGGCCTACGGCGTGCCCAAGTCTGGCCCCTGGCTGATCGAGGCCCTGCGAATCGCCTTCTGGATCTACACCGTGTGCGCTACCCTGCACGCCATTATCTACTACCAGATCATCTTCGTGAACCAGCAGCTGAAGATCACCAACGTGCTGCCCGGCTGGGTGCTGCCTATGTTCCCCGCCATGCTGGTGGGCACCCTGGCTGGCGCCATTGCTAAGACCCAGCCTCCACAGCACGCTTACCCTATGCTGATCGCCGGCCTGTCCTACCAAGGCTTCGGCTTCTTCATGGCCCTGTTCATGTACCCTCTGTACTTCGGCCGACTGCTGACCTCCGGCATTCCCGCCTACCTGTCTCGACCCGCTATGTTCATTGCCGTGGGACCTCCAGCCTTTACCGCTCTGGCTCTGATCAACATGGCCCAGGACGTGCAGGTCACCAAGATCTTCGACGGCTACACCAAGCTGCAGGGCGTCGCCAACCAGGCTCTGATTCCCGACCTGTTCTCTATTATGGCCCTGGTGATGGCCATTTTCCTGTGGGTGCTCGCTTTCTGGTTCTTCGTCATGGCCTTCATTGCCATGATTGACGGACTGCCCCACAACGACTTTCACCTGAACTGGTACGCCAAGGTGTTCCCCAACGTGGGCTTCACCATTGCTACCATCAAGATCGGCGAGCGAATCGACTCTCCCGCCGTGCAGCTGGTGGGAACCGCCATGGCCGCCGTGCTGTTCTTCTCCTGGTGCCTGGTGTTCTTCTGTCACATCAAGGCCTTTCTGGGCCACATGATCTGCTGGCCCGGACGAGATGAGGACGCCCACTAA |


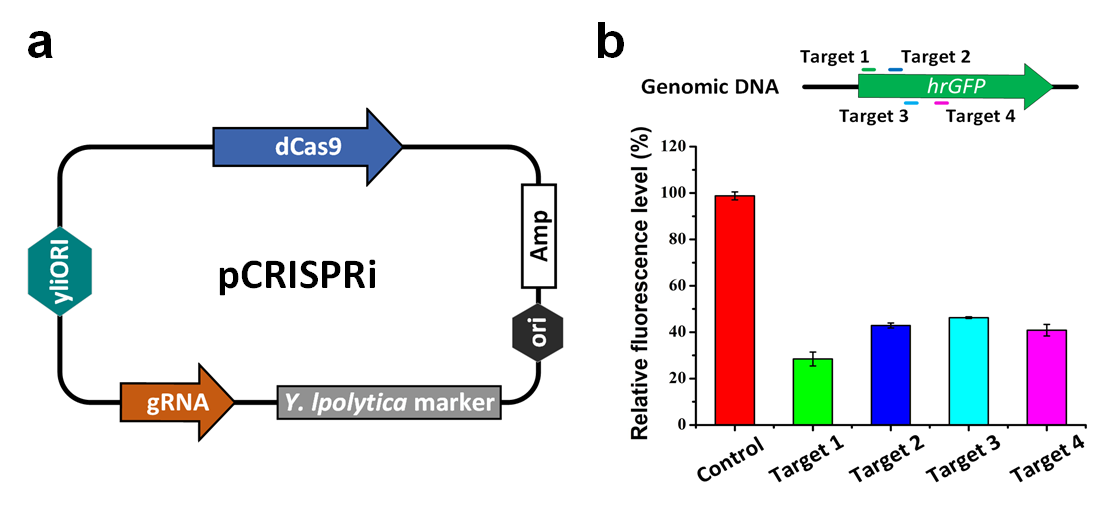


**Figure S1** CRISPR interference (CRISPRi) system in *Y. lipolytica*. (a) Episomal vector pCRISPRi for the simultaneous expression of dCas9 and sgRNA; (b) Functional verification of CRISPRi using *hrGFP* as the reporter gene. 4 different sgRNA targeting sites were designed in the front part of the *hrGFP* gene, its relative expression level could be as low as 27.6% of the control.


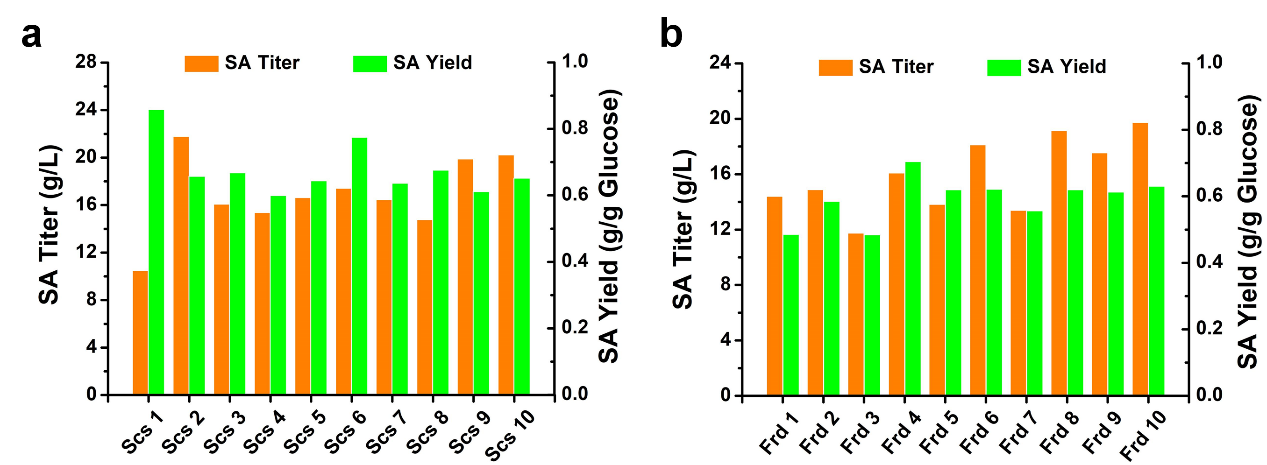


**Figure S2** Screening of transformants with overexpressed *YlScs2* (a) and *TbFrd* (b) through NHEJ- mediated random genome integration for SA production.


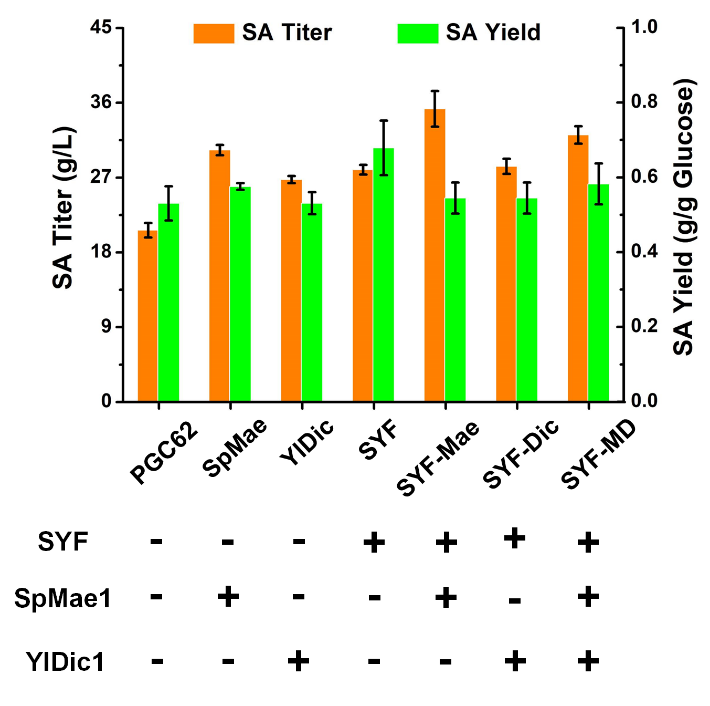


**Figure S3** Comparison of the SA titer and yield between different *Y. lipolytica* engineered strains after 72h fermentation in shaking flasks.

**Reference**

Cui, Z., Gao, C., Li, J., Hou, J., Lin, C.S.K., Qi, Q. 2017. Engineering of unconventional yeast *Yarrowia lipolytica* for efficient succinic acid production from glycerol at low pH. *Metabolic Engineering*, **42**, 126-133.

Cui, Z., Jiang, X., Zheng, H., Qi, Q., Hou, J. 2019. Homology-independent genome integration enables rapid library construction for enzyme expression and pathway optimization in *Yarrowia lipolytica*. *Biotechnology and Bioengineering*, **116**(2), 354-363.

Gao, S., Tong, Y., Wen, Z., Zhu, L., Ge, M., Chen, D., Jiang, Y., Yang, S. 2016. Multiplex gene editing of the *Yarrowia lipolytica* genome using the CRISPR-Cas9 system. *Journal of Industrial Microbiology and Biotechnology*, **43**(8), 1085-1093.
